# Nitrous oxide induced subacute combined degeneration of the spine cord: A case report

**DOI:** 10.1097/MD.0000000000037032

**Published:** 2024-02-09

**Authors:** Shuo Wang, Zhe Li, Yuanyuan Shi, Tianjun Wang, Wei Jin

**Affiliations:** a Department of Neurology, Hebei General Hospital, Shijiazhuang, Hebei, China.

**Keywords:** laughing gas, nitrous oxide, subacute combined degeneration of the spine cord, vitamin B12

## Abstract

**Rationale:**

In recent years, recreational use of inhaled nitrous oxide (N_2_O) is on the increase among young people, accompanied by a corresponding rise in reports about its toxicity. Subacute combined degeneration of the spine cord (SCD) is the typical clinical picture of the nervous system disorder caused by N_2_O intoxication, as a result of metabolic disturbance of vitamin B12.

**Patient concerns, diagnoses, interventions and outcomes:**

We report a 28-year-old female of SCD due to prolonged use of N_2_O, presented with paresthesia and unsteady in walking progressing within 1 month. Symptoms gradually improved with the treatment of intramuscular injections of hydroxocobalamin combined with N_2_O abstinence, and the patient recovered completely with normal neurological examination after 4 months of follow-up.

**Lessons:**

Clinicians should be aware of the clinical features and pathogenesis of SCD caused by N_2_O intoxication in order to lead effective treatment as soon as possible. Recreational N_2_O use should always be considered as an etiology when dealing with patients presented with myelopathy and/or neuropathy suspected of vitamin B12 deficiency.

## 1. Introduction

Nitrous oxide (N_2_O) is a colorless and slightly sweet gas, which was first synthesized in 1772 by Joseph Priestley.^[1]^ It is widely used as a whipped cream foaming agent in the food industry and a fuel booster in the motor industry. Because of its analgesic and anesthetic properties, N_2_O is also used in medicine mainly involved obstetrics, dentistry and emergency. Meanwhile, N_2_O is known as “laughing gas” resulted from its euphoric and relaxed properties since the early 1800s.^[1]^ In the recent years, the recreational use of N_2_O become popular among young people partly because it is cheap and easily accessible.

Nitrous oxide was believed to be harmless until Lassen reported acute aplasia of the bone marrow with prolonged anesthesia with N_2_O in the treatment of tetanus in 1956.^[2]^ Subsequently, Layzer first reported 3 patients developed neurological symptoms after heavy use of N_2_O in 1978^[3]^ and then, in the same year, a total of 15 patients with similar symptoms were summarized.^[4]^ The clinical picture resembled subacute combined degeneration of the spine cord (SCD) and the interference from N_2_O in vitamin B12 metabolism was thought to be the cause of the disorder. Then, reports of similar cases increased and the toxicity of N_2_O was further understood by clinicians. However, this treatable, but relatively rare disease, may not be elicited quickly by clinicians. Here, we describe a case of SCD caused by N_2_O to improve clinicians’ awareness of toxicity of N_2_O abuse.

## 2. Case report

A 28-year-old female was admitted to our hospital with the complaints of numbness in both hands and feet that started 1month ago and then extended to limbs 2 weeks later, accompanied by tingling, tightness and swollen. The patient noticed an occasionally electrical shock-like sensation that run from back to soles when she flexed the neck (Lhermitte sign) and a sudden involuntary muscle contractions in hands accompanied by severe pain occurred spontaneously or triggered by physical contact, lasting for seconds or minutes (painful tonic spams). In addition to paresthesia, the patient presented with weakness in hands and unsteady in walking. The patient was a salesman and had no previous medical history, meanwhile, the patient denied exposure to drugs and the diet was balanced.

**Figure 1.**
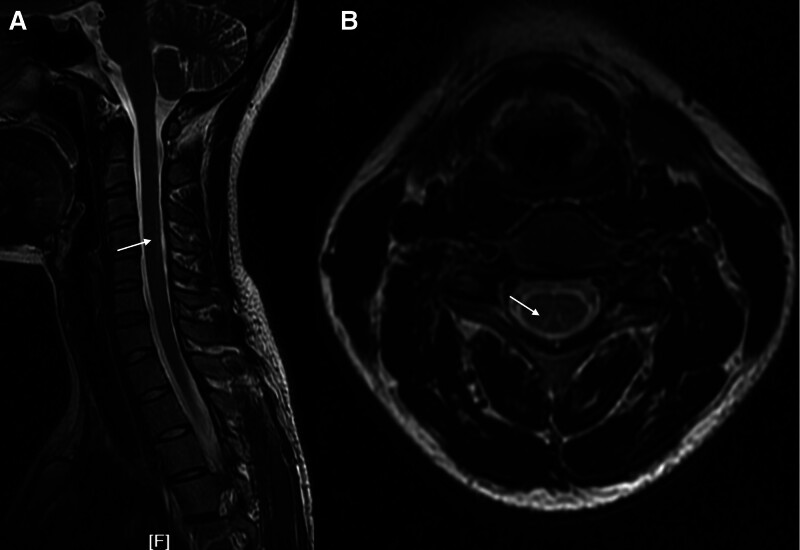
MRI of the cervical spine. The T2 sagittal image (A) shows dorsal column T2 hyperintensity extending over segments C3-C5 (white arrow) and the axial image (B) shows an “inverted V sign” (white arrow). MRI = magnetic resonance imaging.

Neurological examination showed diminished vibratory, touch and position sensation in lower legs, a reduced pain perception in limbs. The tendon reflexes were hypoactive and the plantar response was flexor. The other neurological examination was normal. The magnetic resonance imaging (MRI) of the cervical spine revealed dorsal column T2 hyperintensity extending over segments C3-C5 (Fig. 1A) and the axial images showed an “inverted V sign” (Fig. 1B). An MRI scan of thoracic spine and the brain found no abnormality. Nerve conduction studies demonstrated motor and sensory amplitudes reduction, conduction velocities slowing, and latencies prolongation in lower limbs, consist with mixed axonal and demyelinating neuropathy. Laboratory tests showed no anemia while serum vitamin B12 and folic acid levels were found to be normal. However, the homocysteine showed abnormal with a mild excess of 30 µmol/L.

Therefore, the diagnosis of SCD was elicited and the treatment with hydroxocobalamin injections at 1000 mcg daily was started immediately. The patient ate an adequate diet and had no disease in digestive system, meanwhile the anti-intrinsic factor antibody as well as anti-parietal cell antibody were negative. The etiology of SCD is not clear and the other possible causes are also being considered, mainly including auto-immune disorders and infectious agents. The following anti-aquaporin-4 antibody and anti-myelin oligodendrocyte glycoprotein antibody were negative and there was no evidence of human immunodeficiency virus and Treponema pallidum infection or systemic immune disease. Furthermore, the cerebrospinal fluid analyses were normal.

With a highly suspected exposure of toxicity, the patient mentioned the recreational use of laughing gas for the past 3 years with an average of 10 canisters monthly, up to 300 canisters daily over 1 week before the symptoms aggravated. The patient had experienced transient numbness in limbs after use of N_2_O occasionally in the past 2 years and started supplement with oral vitamin B12 at the advice of friends. But there was no improvement after heavy use of N_2_O on this occasion, and the patient suffered a continuous deterioration. Nitrous oxide-induced SCD was diagnosed and the treatment with hydroxocobalamin injections was continued along with N_2_O abstinence. The patient was discharged from hospital with symptoms partly relieved 2 weeks later. The patient suffered from paresthesia in the extremities and attacks of painful tonic spasms, but decreased in frequency and severity. With partial improvement of symptoms, the frequency of intramuscular injections was reduced to every other day. In the follow-up of 2 month, the patient experienced residual numbness in feet and no painful tonic spasms occurred, then the treatment was discontinued. Symptoms recovered completely with normal neurological examination after 4 months of follow-up.

## 3. Discussion and conclusions

Nitrous oxide is widely used in the food and motor industry, and has the effects of analgesia and anesthesia though the precise mechanism is not well understood. The most recognized mechanism of analgesia is the stimulation of endogenous opioid release in the midbrain and then regulating pain processing in the spinal cord.^[5]^ The noncompetitive inhibition of N-methyl-D-aspartate receptors leading to inhibition of excitatory glutamatergic neurotransmission may be the possible mechanism of anesthesia.^[6,7]^ However, the euphoric and hallucinogenic properties, probably due to inhibition of N-methyl-D-aspartate receptors, make it an addictive drug and could be toxic to humans with unreasonably heavy use.

The majority of accessible form of recreational use of N_2_O is canister, usually containing 8g of gas, and the pressured gas was stored in a volume of 10 cm^3^. When inhaled, the gas is released into a balloon, equivalent to 4~8L gas at normal pressure. A heavy user may use cylinder (1 cylinder is approximately equal to 75 canisters) to consume large amounts. After inhalation, euphoria occurs in seconds and then fades away within minutes without hangover. The striking but temporary effect may induce users to consume without cease, ranging from a couple to hundreds in an occasion.^[8,9]^ There appeared to be an increase in the adverse effects corresponding with the increase use of N_2_O,^[10]^ mainly involving accidents and chronic symptoms of nervous system.

Accidents associated with recreational N_2_O use mainly include skin and mucosa damage resulted from hypothermia because the pressured N_2_O is extremely cold. Seizures or arrhythmia could occur in patients with certain comorbidities, such as cardiovascular disease, mainly resulted from hypoxia. The most serious accident, asphyxia, mainly due to a specific inhalation method, for example, a bag over the head or in an enclosed car full of N_2_O.^[11]^ Combined with alcohol or other drugs would increase the risks. At the same time, people may complain about the symptoms of nausea, hallucination, confusion, behavioral disinhibition after inhalation of N_2_O, even transient numbness, tingling, unsteady walking, impairment of memory, which may progress to severe and chronic conditions when prolonged N_2_O use is continued. The typical clinical picture is SCD, as a result of metabolic disturbance of vitamin B12.

**Figure 2.**
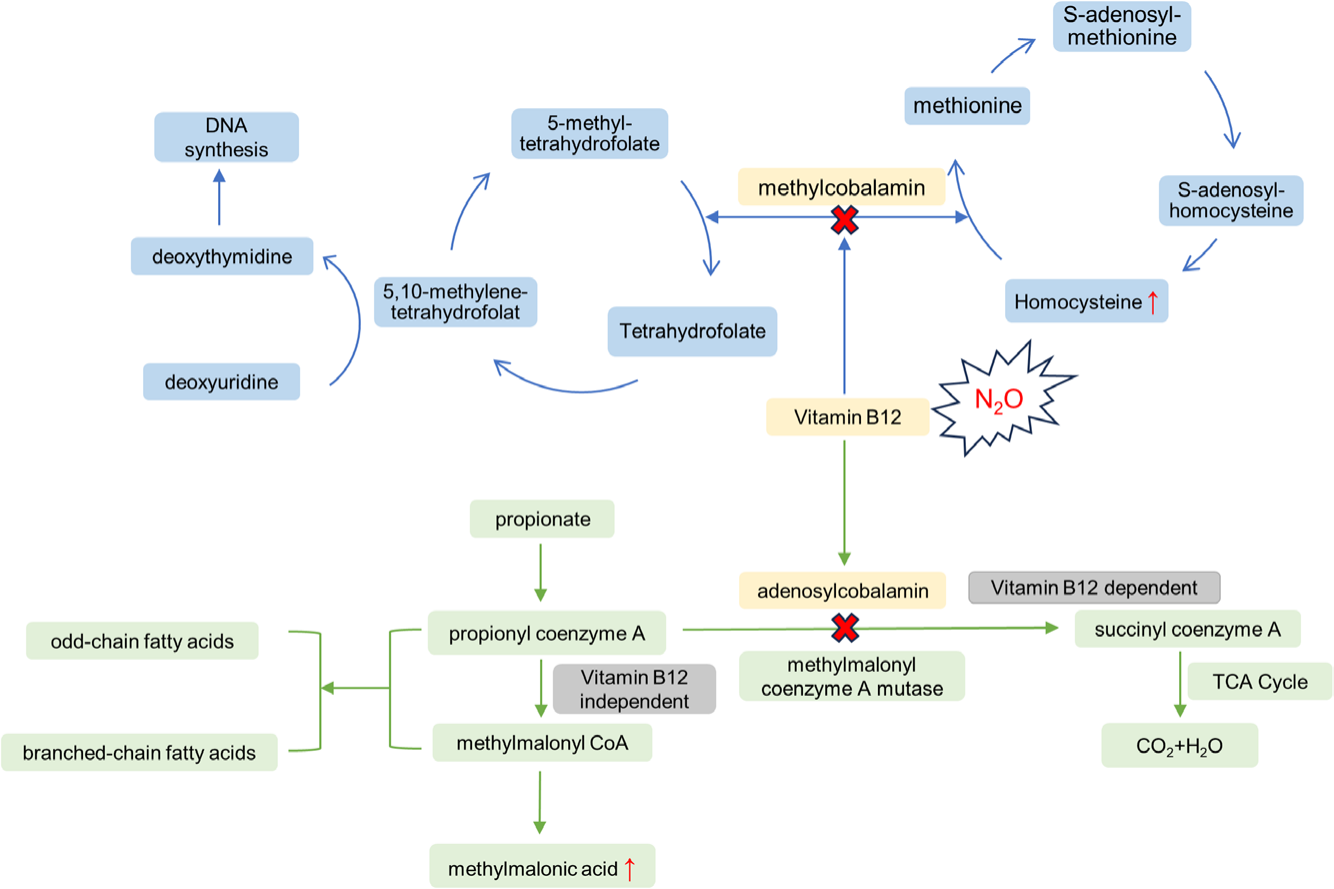
The interference from N_2_O in vitamin B12 metabolism. Vitamin B12 (cobalamin) acts as a coenzyme in 2 enzymatic reactions in the biologically form of methylcobalamin and adenosylcobalamin respectively. N_2_O irreversibly oxidizes cobalamin, leading to a functional vitamin B12 deficiency. Methylcobalamin participates the metabolism of homocysteine to methionine as well as the conversion of 5-methyltetrahydrofolate to tetrahydrofolate. With methylcobalamin deficiency, there is a corresponding accumulation of homocysteine, leading to demyelination and megaloblastic changes in the bone marrow due to methyl-deficiency and impaired DNA synthesis respectively. Adenosylcobalamin catalyzes the conversion from methylmalonyl CoA to succinyl coenzyme A. The inhibition of this vitamin B12 dependent process resulted in an elevation of methylmalonic acid and disturbance of fatty acid in myelin, further leading to demyelination of neurons. N2O = nitrous oxide.

Vitamin B12, also called cobalamin, acts as a coenzyme in 2 enzymatic reactions in the biologically form of methylcobalamin and adenosylcobalamin respectively. N_2_O irreversibly oxidizes cobalamin, leading to a functional vitamin B12 deficiency. Methylcobalamin participates the formation of methionine, converting homocysteine to methionine as well as the conversion of 5-methyltetrahydrofolate to tetrahydrofolate (THF) in this recycling action (Fig. 2). Methionine is then converted to S-adenosylmethionine, a main methyl donor, participating the process of methylation of myelin basic protein. It had been suggested that a highly methylatable myelin basic protein structure is crucial in the integrity and maintenance of myelin, whereas, the methyl-deficiency is linked to demyelination. With methylcobalamin deficiency, there is a corresponding decrease in methionine/S-adenosylmethionine and an accumulation of homocysteine, meanwhile, the conversion of THF is blocked. THF is usually converted to 5,10-methylenetetrahydrofolate, required for the de novo synthesis of DNA involving the conversion of deoxyuridine to deoxythymidine. The impaired DNA synthesis could lead to megaloblastic changes in the bone marrow.^[12]^ Adenosylcobalamin acts as a coenzyme of methylmalonyl coenzyme A mutase, catalyzing the conversion from methylmalonyl CoA to succinyl coenzyme A. When this vitamin B12-dependent process is blocked, there would be an accumulation of methylmalonyl CoA, along with the elevated methylmalonic acid and propionyl CoA. An abnormal increase in propionyl CoA and methylmalonyl CoA would lead to a corresponding increase in odd-chain and branched-chain fatty acids, which accumulate in membrane lipids of nervous tissue. Changes in composition of fatty acid in myelin may destroy its integrity and cause demyelination, resulted in impaired nervous system functioning ultimately.^[13]^

Therefore, the metabolic disturbances were thought to be the pathogenesis of vitamin B12 deficiency myelopathy and/or neuropathy, and determine the clinical pictures of the disorder. The initial symptom is usually symmetrical paresthesia in the distal lower limbs, together with terminal upper limbs sometimes.^[4]^ Patients tend to present with gait ataxia and unsteadiness on walking with the prolonged use of N_2_O.^[14]^ The less common symptoms include weakness, Lhermitte sign, painful tonic spams, psychiatric complaints, impotence, spastic bladder and bowel symptoms.^[4,15]^ When suspected, serum vitamin B12 level test is the first step, and the results could be lower, sometimes normal or even higher than the standard range, partly due to the supplement with vitamin B12 before the examination. However, homocysteine and methylmalonic acid are found to be elevated in almost patients, which are more specific than serum vitamin B12 and represent the functional vitamin B12 deficiency.^[14,16,17]^ MRI scans of spinal cord is a useful examination in the diagnosis of myelopathy and confirm the etiologies. The most common abnormality in spinal MRI was T2 hypersignal in dorsal column involving the C3-C5 segments, with “inverted V” or “rabbit ears” sign in axial images,^[14,15]^ whereas, the thoracic cord is less involved compared with the classic SCD.^[18]^ Furthermore, the mixed axonal and demyelinating neuropathy is the most common pattern of nerve conduction studies of N_2_O-induced neuropathy, which has been reported in the research,^[19]^ consistent with our patient. Therefore, the patient was eventually diagnosed as N_2_O induced SCD with the evidence of involvement of spinal cord and neuropathy, however, the concealment of inhale of N_2_O increases the difficulty of diagnosis.

As soon as the diagnosis of SCD is suspected, patient should receive daily intramuscular injections of 1000 mcg hydroxocobalamin immediately. When N_2_O is identified as the cause, N_2_O abstinence should be carried out combined with patient education. Treatment usually lasts for at least 2 weeks and then gradually reduced until the patient symptoms no longer worsen.^[17]^ The majority of patients could have a good recovery over a period of weeks to months.

In conclusion, when dealing with patients with myelopathy and/or neuropathy suspected of vitamin B12 deficiency, recreational N_2_O use should always be considered as an etiology. Clinicians should be aware of the clinical features and pathogenesis of the disorder in order to lead effective treatment as soon as possible. Regular follow-up is essential for a better prognosis and discovering the other possible causes of vitamin B12 deficiency. Besides, patient education needs to be strengthened to make patients aware of the toxicity of N_2_O and stay away from N_2_O abuse.

## Author contributions

**Conceptualization:** Shuo Wang, Wei Jin.

**Funding acquisition:** Tianjun Wang.

**Investigation:** Tianjun Wang.

**Project administration:** Yuanyuan Shi.

**Resources:** Zhe Li.

**Supervision:** Wei Jin.

**Visualization:** Yuanyuan Shi.

**Writing – original draft:** Shuo Wang, Zhe Li.

**Writing – review & editing:** Shuo Wang, Zhe Li, Wei Jin.
